# Supplementary material for: Content-rich biological network constructed by mining PubMed abstracts
Source: BMC Bioinformatics. 2004 Oct 8;5:147. doi: 10.1186/1471-2105-5-147 (PMC528731; doi:10.1186/1471-2105-5-147)
Supplement: Additional File 2 — The original results of the above study (non-essential files are deleted to keep the file size under the limit set by BMC bioinformatics). [file 1471-2105-5-147-S2.bz2 › chilibotAdditionalFile2/dip05/619ID10673434E11003/html/FNTA_FNTB.html]

 


 **FNTA** and **FNTB** 
  
Found 1 abstracts in PubMed, retrieved 1.  
 

 What does Google say? 
 PDF only 
| .edu only 

---

**Interactive relationship** (e.g. stimulation, inhibition, etc)

**Non-interactive relationship** (e.g. studied together, co-existance, homology, etc.)

- cDNA cloning of the two subunits of human CAAX farnesyltransferase and chromosomal mapping of  **FNTA**  and  **FNTB**  loci and related sequences.  Ref: 8276393 Genomics, 1993
- The functional genes for the alpha and beta subunits of human farnesyltransferase gene symbols,  **FNTA**  and  **FNTB**  were localized to human chromosome bands 8p22 q11 and 14q23 q24, respectively, by Southern blot hybridization and PCR analyses of panels of human x Chinese hamster somatic cell hybrid lines and by fluorescence chromosomal in situ hybridization.  Ref: 8276393 Genomics, 1993
